# Systematic evaluation of sericin protein as a substitute for fetal bovine serum in cell culture

**DOI:** 10.1038/srep31516

**Published:** 2016-08-17

**Authors:** Liyuan Liu, Jinhuan Wang, Shengchang Duan, Lei Chen, Hui Xiang, Yang Dong, Wen Wang

**Affiliations:** 1 State Key Laboratory of Genetic Resources and Evolution, Kunming Institute of Zoology, Chinese Academy of Sciences, Kunming 650223, China; 2 Kunming College of Life Science, University of Chinese Academy of Sciences, Kunming 650223, China; 3 Kunming University of Science and Technology, 727 South Jingming Road, Chenggong District, Kunming 650500, China; 4 South China Normal University, Guangzhou, 510631, China

## Abstract

Fetal bovine serum (FBS) shows obvious deficiencies in cell culture, such as low batch to batch consistency, adventitious biological contaminant risk, and high cost, which severely limit the development of the cell culture industry. Sericin protein derived from the silkworm cocoon has become increasingly popular due to its diverse and beneficial cell culture characteristics. However, systematic evaluation of sericin as a substitute for FBS in cell culture medium remains limited. In this study, we conducted cellular morphological, physiological, and transcriptomic evaluation on three widely used mammalian cells. Compared with cells cultured in the control, those cultured in sericin-substitute medium showed similar cellular morphology, similar or higher cellular overall survival, lower population doubling time (PDT), and a higher percentage of S-phase with similar G2/G1 ratio, indicating comparable or better cell growth and proliferation. At the transcriptomic level, differentially expressed genes between cells in the two media were mainly enriched in function and biological processes related to cell growth and proliferation, reflecting that genes were activated to facilitate cell growth and proliferation. The results of this study suggest that cells cultured in sericin-substituted medium perform as well as, or even better than, those cultured in FBS-containing medium.

The development of biopharmaceuticals has created an unprecedented increase in demand for cell culture products. The global cell culture industry, which occupies an important position in biological sciences, is a major component of the biopharmaceutical market^1^, and is expected to reach $18,630.7 m by 2020, up from $11,310.9 m in 2015^2^. To date, animal-origin materials, such as fetal bovine serum (FBS), have been the predominantly used ingredients in cell culture media. Although FBS provides attachment and growth factors, as well as the nutritional and physiochemical compounds required for cell maintenance and growth^3^,^4^, it still has numerous deficiencies and disadvantages. Firstly, strict quality control is difficult due to low batch to batch consistency from bovine fetuses, causing serious problems in scientific research and industries based on cell culture. Secondly, preparation of FBS involves complicated procedures, and can be easily contaminated by microbes such as Mycoplasma. Thirdly, ethical issues exist in the acquisition of bovine serum, which requires many cattle and increases environmental pollution from gas emissions. Increasingly, concerns have been raised about FBS safety and its deficiencies in cell culture^5^,^6^, highlighting the need for FBS substitutes^7^. However, although considerable effort has been made in the development of diverse FBS-free media, comprehensive exploration and evaluation of such media is still strongly desired^8^,^9^.

Sericin is a major component of silkworm silk protein, helping to envelop the fibroin fiber during cocoon formation by cementing the fibrous core protein together^10^. Traditionally, sericin is discarded during filature production in the silk industry, forming a substantial constituent in waste water and resulting in serious environmental pollution^11^. On the other hand, sericin is reported to be a mitogenic factor involved in better developmental competence of mammalian zygotes^12^, as well as better cell proliferation^13^,^14^,^15^ and attachment^16^,^17^, and has been used in the culture of Sf 9 insect cells^18^, human skin fibroblast cells^19^, human marrow stromal cells (hMSCs), endothelial cells, T-lymphocytes, and hybridomas^20^,^21^. To date, however, systematic evaluation of sericin as a component of serum-free media for the cultivation of different kinds of cells is limited^22^, which has greatly hampered the wide acceptance and application of FBS-free sericin medium in the cell culture industry.

In this study, we used three cell lines, which represent the most commonly used cell types in cell culture, to evaluate the performance of sericin as a FBS medium substitute. The three kinds of cells were Chinese hamster ovary (CHO) cells, which represented fibroblast-like cells; African green monkey kidney (MARC-145) cells, which represented epithelial cells; and, the HeLa cell line, which represented tumor cells. The CHO cell line is one of the most effective and successful expression systems for exogenous proteins and the most commonly used mammalian host for industrial production of recombinant protein therapeutics^23^,^24^,^25^. The MARC-145 cell line is epithelial-like and a good host for the reproduction of the porcine reproductive and respiratory syndrome (PRRS) virus due to its susceptibility^26^,^27^. The HeLa cell line is a very important tumor model for cell biology, cancer, virus propagation, biosynthesis, and anti-tumor mechanism research^28^,^29^,^30^.

We compared the morphological, physiological, and transcriptomic features between cells cultured in sericin-substituted medium and those cultured in conventional FBS-containing medium, and found the former performed as well as or even better than the latter in terms of all cellular features mentioned above, thus providing a strong reference for boosting the application of FBS-free media in the cell culture industry.

## Results

### Cellular morphology and overall survival

**Figure 1:**
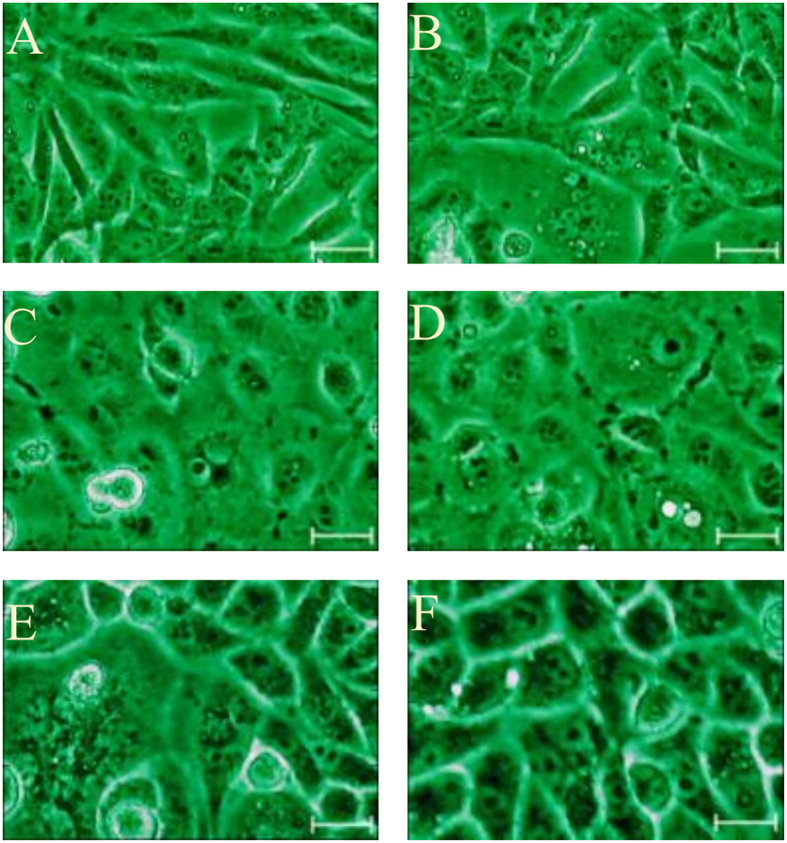
Morphology of CHO cells, MARC-145 cells and HeLa cells in control medium and sericin-substituted medium at 80–90% cell confluence. No significant difference of cell morphology was observed in cells cultivated in the control medium and in the sericin-substituted medium. (**A,B**) CHO cells; (**C,D**) MARC-145 cells; (**E,F**) HeLa cells. (**A,C,E**) control medium (10% FBS); (**B,D,F**) sericin-substituted medium (30 μg/ml sericin protein). Scale bars represent 40 μm.

We identified every cell in every image in the experiment using cell-image analysis software (CellProfiler). The three kinds of cells grew similarly well in both the sericin-substituted and control media, and also showed normal cell morphology (Fig. 1). In detail, CHO cells cultivated in sericin-substituted medium exhibited a spread, fibroblast-like morphology with extensive cell-cell contacts, the same as that of cells cultivated in the FBS-containing medium (Fig. 1A,B). The MARC-145 cells were epithelial-like and grew similarly well in both media (Fig. 1C,D). The typical cell morphology of the HeLa cells (Fig. 1E,F), specifically a subconfluent monolayer of cells with an unoccupied surface and cell boundary and condensed nuclear chromatin, was shown *in vitro* in both media. The unaltered cellular morphology suggests that sericin well supported the growth of the three kinds of cells. Furthermore, no significant differences in cell morphology were observed between cells cultivated in either media based on cell size, shape, and outline (Supplemental Fig. S1).

**Figure 2:**
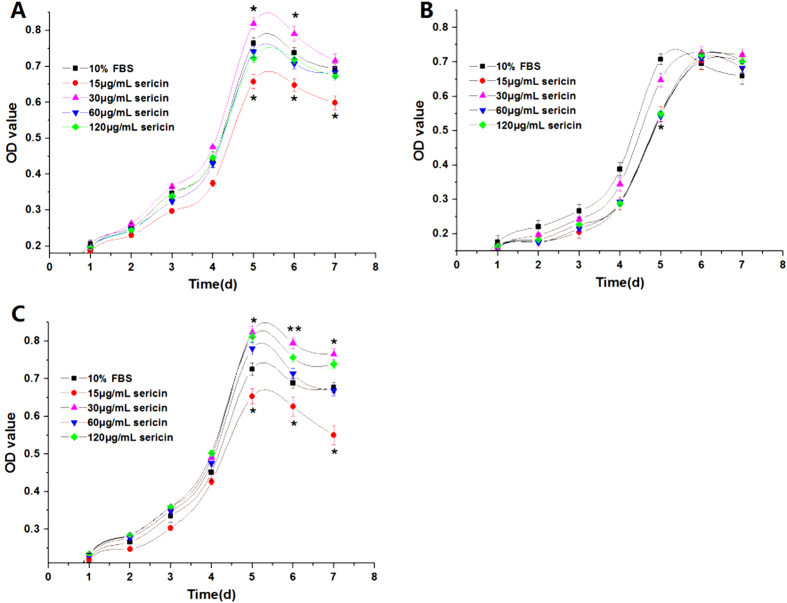
The metabolic activity curve of cells. The cell survival curve by MTT assay for three cell lines in its respective culture medium at different time points. The longitudinal axis Y displays the cell OD value of MTT, which refers to overall cell survival. Data are shown as the Mean ± SD (n = 4). *P < 0.05 significant difference with respect to control medium values; **P < 0.01 significant difference with respect to control medium values. A. CHO cells; B. MARC-145 cells; C. HeLa cells.

After morphological observation, cell overall survival was examined by thiazolyl blue tetrazolium bromide (MTT) assay. Based on different concentrations of sericin, 30 μg/ml was found to be optimal for all three cell lines (Fig. 2). Specifically, except for the low sericin dose (15 μg/ml), CHO cells showed similar overall survival in both sericin-substituted and control media (Fig. 2A). The HeLa cells showed significantly higher overall survival in the sericin-substituted media (Fig. 2C). Although the MARC-145 cells in the 15 μg/ml, 60 μg/ml, and 120 μg/ml sericin-treated media showed lower overall survival compared with those in the control, cells still showed comparable overall survival under the 30 μg/ml sericin treatment (Fig. 2B).

### Cell cycle distributions and cell replicative capacity

**Figure 3:**
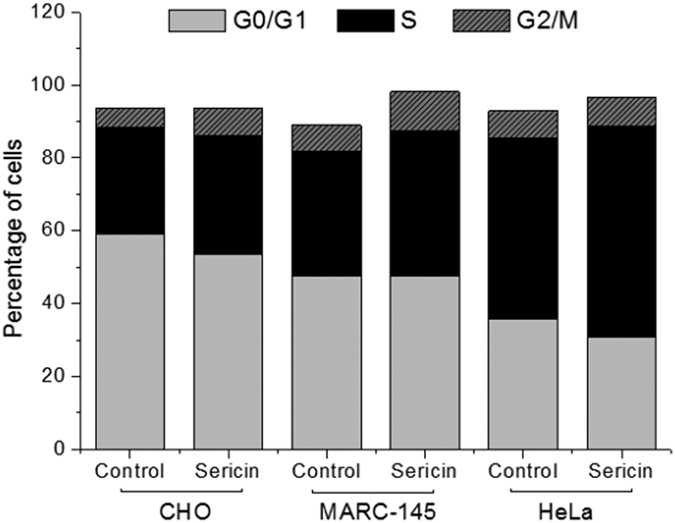
Cell cycle distribution in the sericin-substituted medium and control medium for the three cell lines. Cell cycles of the three cell lines were aggregation in the S phase in the sericin-substituted medium. However, cells in the sericin medium showed a non-significant increase in the percentage of S phase accompanied by a proportional decrease of G0/G1 and G2/M phases compared with cells in the control medium The number of cells in the G0/G1-, S- or G2/M-phase is given as percentages of the total cell population.

**Figure 4:**
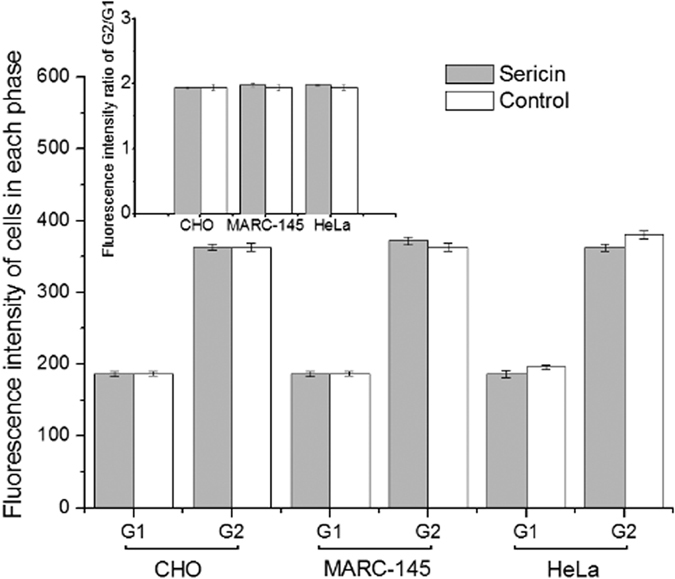
Cellular DNA content in the experimental and control medium for the three cell lines. Data are shown as the Mean ± SD values for the three cell lines (n = 4). There was no significant difference in cellular DNA content. Control: control medium (10% FBS); Sericin: sericin-substituted medium (FBS-free, 30 μg/ml sericin protein).

For cell cycle distributions, cells in the sericin medium showed a non-significant increase in the percentage of S phase accompanied by a proportional decrease of G0/G1 and G2/M phases compared with cells in the control medium (Fig. 3, Supplemental Fig. S2). These results indicated that there was a similar (or greater) number of cells active in DNA synthesis in the sericin medium than in the control medium. Meanwhile, we did not detect any noticeable peak value ratio shift in G0/G1 and G2/M phases cells between the cells cultured in either media (Fig. 4), suggesting no deleterious distortion in cellular genetic programming^31^.

**Figure 5:**
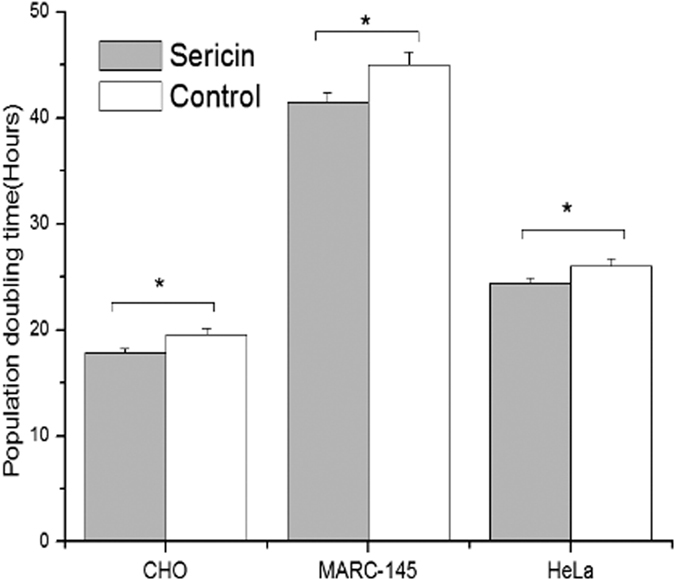
Population doubling time of three types of cells in sericin-substituted and control medium. Data are shown as the Mean ± SD values for the three cell lines (n = 4). Control: control medium (10% FBS); Sericin: sericin-substituted medium (FBS-free, 30 μg/ml sericin protein). Asterisks indicate significant difference (*P* < 0.05) by two-tailed paired Student *t*-test.

Cellular replicative capacity was analyzed by the measurement of population doubling time (PDT). The PDT values of all three cell types in the sericin-substituted medium were significantly lower than those in the control medium, suggesting that the proliferation rate of cells was higher in the sericin-substituted medium (Fig. 5). The above results indicate that cells in serum-free medium containing 30 μg/ml of sericin showed similar or better cell cycle and cell replicative capacity than that observed in the control medium.

### Comparisons of gene expression profiles

**Figure 6:**
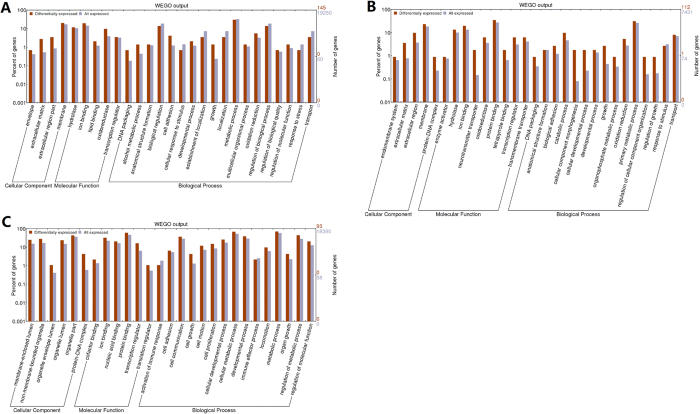
Enrichment analysis. GO enrichment analysis of DEGs for the three cells cultivated in sericin-substituted and control medium. X-axis: function items. Y-axis (left): gene percents. Y-axis (right): number of genes. (**A**) CHO cells, with 19,250 genes that have GO annotations, 145 DEGs showed significant enrichment difference *(P* < 0.05, *χ*^*2*^test); (**B**) MARC-145 cells, with 7,431 genes that have GO annotations, 112 DEGs showed significant enrichment difference (*P* < 0.05, *χ*^*2*^test); (**C**) Hela cells, with 18,390 genes that have GO annotations, 93 DEGs showed significant enrichment difference (*P* < 0.05, *χ*^*2*^test).

For gene expression profile analysis, we generated more than 43 million reads per sample. In detail, a total of 49.84 million reads (6.23 Gb, control medium) and 50.37 million reads (6.30 Gb, sericin-substituted medium) were obtained for the CHO cells; 43.34 million reads (5.4 Gb) and 44.22 million reads (5.5 Gb) were obtained for the MARC-145 cells; and, 50.32 million reads (6.29 Gb) and 65.42 million reads (8.18 Gb) were obtained for the HeLa cells, respectively. The majority (77.8% and 81.7% for CHO cells; 75.4% and 72.6% for MARC-145 cells; 81.4% and 78.4% for HeLa cells) could be mapped uniquely to the respective reference genomes. Furthermore, we found a total of 145, 112, and 93 differentially expressed genes (DEGs) between CHO, MACR-145, and HeLa cells, respectively, cultured under the two culture conditions. The number of DEGs accounted for 1.01%, 0.74%, and 0.77% of all detected genes in the corresponding genomes, respectively. Given that there are more than 20,000 genes in mammalian genomes, the number of DEGs found in the present study were relatively low, suggesting that substitution of FBS with sericin did not cause serious changes in the cells’ gene expression profiles. Moreover, GO enrichment analyses showed that the DEGs were over-represented in molecular functions such as energy substance binding, enzyme regulation, and structural molecules, as well as biological processes such as DNA packing, cell activation, cell adhesion, cell proliferation, development processes, biological regulation, and metabolic processes (Fig. 6). Specifically, upregulated genes were associated with cell energy metabolism and DNA replication, such as cellular respiration (*HMGCS1, TXNIP* and *ABCA13*) and cell cycle progression (*CKS1, RMRP, FN1* and *MFSD8*) (Supplemental Table S1, S2 and S3), whereas downregulated genes were associated with extracellular matrix transportation, ribonucleoprotein complex, and cell growth suppression such as inhibition of DNA binding (*ID1, ID2* and *ID3*), transcriptional activation ability, and fibrinolysis (Supplemental Table S1 and S2). In addition, we generated a Venn diagram to visualize the DEG overlap for a three-way comparison. As shown in Supplementary Fig. S3, the proportions of cell line-specific DEGs were 30.3% in CHO cells, 16.1% in MARC-145 cells, and 11% in HeLa cells, indicating fairly large overlap. The high DEG overlap suggests that the RNA-seq analysis was reliable and consistent. Furthermore, consistent with the observations from the phenotypic and metabolic states of cells, genes associated with these functions and related biological processes were activated to facilitate cell survival and proliferation.

## Discussion

This study undertook a systematic assessment of sericin as a substitute for FBS in cell culture by investigating cell morphology, cell overall survival, cell cycle and proliferation capacity, as well as transcriptomic analysis.

Morphological observation is commonly used in cell status assessment, and has been applied to evaluate the effect of serum-free or serum-reduced media on cell culture in several studies^32^,^33^. However, these previous studies have been limited to investigating cellular survival. In the current experiment, the effect of sericin-substituted medium was assessed in regards to cell morphological comparison, cell cycle, and PDT assessment. Our results suggest that sericin can replace FBS in the cell culture process with no significant differences in cell morphology, consistent with earlier experiments related to sericin and morphology of cultured cells^34^,^35^. Furthermore, in regards to cell cycle and PDT, sericin was found to promote cell growth and shorten PDT, which has been reported before in the process of fibroblast culture^36^.

Previous application of serum-free media has often used expensive growth-related cytokines, such as fibroblast growth factor, leukemia inhibitory factor and transforming growth factor, rather than sericin^37^,^38^,^39^,^40^. Takahashi *et al*.^41^ first tried sericin and found that supplementation with SerD improved the serum-free culture of insect cells. Since then, sericin has been used as a constituent of cell culture to promote the proliferation of cells in serum-free media^42^,^43^,^44^. However, it has not been treated as an entire substitute for FBS, and no comprehensive evaluation of its effectiveness as a serum-free medium has been conducted. For example, Miyamoto *et al*.^43^ used sericin together with dimethylsulfoxide as a supplement to culture and cryopreserve human hepatocytes, which improved cell-attaching capability and viability in the serum-substitute medium. Thus, it is difficult for these methods to obtain or clarify the precise molecular mechanism for the promotion of cell growth.

By optimizing sericin concentration using a series of gradients, we successfully applied sericin as a complete substitute for FBS. Our preliminary results showed that at the optimal concentration sericin possessed a better capacity to promote cell growth and reproduction, compared with the growth-promoting activity of different sericin extraction methods^36^, factor addition methods^44^ and partial sericin substitution methods^45^, by measuring cell overall survival and PDT.

We also measured cellular DNA content and analyzed the cell cycle, which have not been investigated in previous research. We found a non-significant increase in the percentage of S phase cells accompanied by a proportional decrease of G0/G1 and G2/M phase cell populations, especially in the HeLa cells. This showed an aggregation in the S phases of the three cells in the sericin-substituted medium, thus suggesting an increased proliferative potential^46^,^47^. It is notable that the promotion of the cell cycle did not cause deleterious distortion in cellular genetic programming^48^. Given that deregulation of the cell cycle will lead to abnormal proliferation of DNA-damaged cells and evasiveness of apoptosis^49^, the results of the present study clearly showed that sericin-substituted medium could promote the cell cycle, without apoptosis or cell damage on the tested cells.

The above cell cycle and cell replication capacity assay results illustrated that the cell division programs of the three kinds of cells were highly activated in the sericin-substituted medium compared with those in the control medium, without causing unfavorable changes (apoptosis or damage) following promotion of the cell cycle. In this study, for the first time, we evaluated the cell status in sericin-substituted medium at the transcriptome level, which is a promising tool for providing molecular cues for the promotion of cell growth and proliferation. Consistent with the observations from the cell phenotypic assay, genes associated with growth functions and involved in the related biological processes were activated to facilitate cell growth and proliferation. The upregulated genes were associated with cell energy metabolism and DNA replication, such as cellular respiration. The importance of DNA packing and cell activation in cell differentiation is well recognized^50^,^51^,^52^, and they benefit from integration of cell-cell and cell-matrix interactions. Similarly, biological dynamics of cell adhesion affect the nature of cell-cell and cell-matrix interactions that regulate events such as cell proliferation, differentiation, and tissue formation^53^,^54^. Cell adhesion molecules are important for interfacial interactions that support transduction of signals between cells and the extracellular matrix^55^,^56^. The downregulated genes were mainly extracellular matrix transporters and were involved in cell growth suppression processes, such as inhibition of DNA binding (*ID1, ID2* and *ID3*), transcriptional activation ability, and fibrinolysis. Suppression of the expression of *ID1, ID2*, and *ID3* genes is known to release the inhibition of the DNA binding process^57^. Overall, GO enrichment revealed that the DEGs were enriched in the processes of promoting cell growth and proliferation, further supporting our cell morphological, cell cycle and PDT assessment. Ten DEGs of the MACR-145 cells were selected for validation by quantitative real-time polymerase chain reaction (qRT-PCR). All genes showed consistent trends between the RNA-seq and qRT-PCR trials (Supplemental Fig. S4).

We conducted a systematic assessment of sericin as a substitute for FBS in cell culture. The growth state of cells was as good as or even better in the sericin-substituted medium than that in the traditional FBS medium. Our findings not only provide conclusive evidence for the application of sericin as a replacement for FBS, but will also assist in future understanding of sustainable development in both the cell culture and silk industries.

## Methods

### Cell lines and culture conditions

The CHO, MARC-145, and HeLa cells were cultivated at the Kunming Cell Bank of the Chinese Academy of Sciences. By preliminary testing, we determined the suitable inoculation densities of each cell line, which were 2.5 × 10^5^ for CHO cells, 9.6 × 10^5^ for MARC-145 cells, and 6.7 × 10^5^ for HeLa cells per well in 6-well culture plates, respectively. The three cell lines were then inoculated at the suitable inoculation densities for further culturing. The CHO cells were seeded in Dulbecco’s Modified Eagle’s Medium (DMEM) supplemented with antibiotics (100 U/ml penicillin, 100 μg/ml streptomycin), 0.3 mM L-proline, 25 mM methionine sulfoximine, 2 mM L-glutamine, 20 mM HEPES, and 0.25% peptone. They were maintained in 6-well culture plates at 37 °C, 70% relative humidity, and 5% CO_2_/95% air atmosphere. For MARC-145 and HeLa cells, standard cell culture techniques were used, with cells cultured in DMEM containing 4.5 g/L glucose, 2 mM L-glutamine, 100 U/ml penicillin, and 100 μg/ml streptomycin and incubated at 37 °C and 5% CO_2_/95% air atmosphere. Upon the basic cell culture conditions of the three cell lines, we established sericin-substituted and control FBS treatments, respectively. For sericin-substituted treatment, sericin protein (Sigma, St. Louis, MO, USA) was added to the basic cell culture medium at designated concentrations (15 μg/ml, 30 μg/ml, 60 μg/ml, or 120 μg/ml). For the control, FBS (Sigma, St. Louis, MO, USA) was added to the basic cell culture medium to a final concentration of 10%. All cell culture reagents were purchased from Invitrogen (Carlsbad, CA, USA) unless indicated otherwise. Based on cell growth and overall survival assessment, the sericin-substituted medium at the optimal sericin concentration was used for other assessments. Cells at the logarithmic growth phase were used for the evaluation of cell morphology, overall survival rate, cell cycle and PDT. In addition, transcriptomic analyses were conducted on the sericin-substituted and control groups for each cell line.

### Cell growth assessment

For both the sericin-substituted and control groups, cell growth was assessed by investigating cell overall survival and morphology. Cell overall survival was assessed by thiazolyl blue tetrazolium bromide (MTT) (Sigma, St. Louis, MO, USA) assay, based on the activity of mitochondrial dehydrogenase enzymes in cells^58^. Metabolically active cells can metabolize MTT to insoluble formazan by dehydrogenases, while the color of the solution changes from yellowish to violet-blue. Formazan is soluble in organic solvents and can be estimated by spectrophotometry. Formazan quantity is proportional to the number and overall survival of cells. Under the same cell number and volume, cell overall survival was determined by optical density (OD) at 570 nm, with background subtraction at 650 nm, using a microplate reader (Infinite^®^ 200 PRO NanoQuant, Tecan, Switzerland). From day 1 to day 7, cells were subject to MTT assay daily with four technical repetitions each time.

Cell images were recorded with a Leica DMI4000B microscope (Leica, Wetzlar, Germany) at 80–90% cell confluence during cell cultivation. Cell identification and cell image analyses were carried out using open source CellProfiler software^59^. Cell outline and size data were collected in the logarithmic phase.

### Cell proliferation assessments

#### Flow cytometric analysis of cell cycle distribution

As mentioned above, medium substituted with 30 μg/ml of sericin, which was the optimal concentration for all three cell lines, was used for assessment of cell cycle and cell replicative capacity. Cell cycle distribution was investigated by evaluating the DNA content (fluorescence intensity) of different cell phases. Cellular DNA content was evaluated using propidium iodide (PI) (Sigma, St. Louis, MO, USA) staining of permeabilized cells using flow cytometry (BD FACS Calibur, New Jersey, USA) according to a modified method^60^. Briefly, after 80–90% cell confluence during cell cultivation, the cell layer was harvested in solution containing 0.25% (w/v) trypsin and 0.53 mM EDTA to remove all traces of culture medium compositions, then washed with ice-cold phosphate-buffered saline (PBS), fixed in 75% ethanol and stored at −20 °C overnight. Cells were then collected by centrifugation (2500 rpm at room temperature for 3 min) and resuspended in 20 mg/ml of PI solution with 0.5 U RNase A and 0.02% Triton X-100 (Sigma, St. Louis, MO, USA), then incubated in the dark at 37 °C for 30 min. A total of 2 × 10^4^ cells/sample were analyzed by flow cytometry using an instrument equipped with Cell Quest software, with four technical repetitions performed each time. The percentages of PI-stained cells in G0/G1, S, and G2/M phases were recorded, and G1 and G2 peak fluorescence intensities and the G2/G1 ratios were evaluated.

#### Population doubling time (PDT) evaluation on cell replicative capacity

Cell replicative capacity was evaluated by PDT^61^, which was estimated using an online population doubling calculator^62^. This analysis was performed every day, with four technical repetitions conducted each time during the exponential phase.

### RNA-seq

#### RNA-seq library construction and sequencing

Cells cultivated in the sericin-substituted and control media were harvested on day 7 (3–7 successive cell cycles) for RNA-seq. Total RNA was extracted using TRIzol (Invitrogen, Carlsbad, CA, USA) according to the manufacturer’s instructions. Prior to reverse transcription, RNA was treated with DNase I (Invitrogen, Carlsbad, CA, USA) to remove contaminating genomic DNA. Both RNA quantity and quality were determined using a Qubit 2.0 fluorometer (Invitrogen, Carlsbad, CA, USA)^63^. The integrity of the RNA samples was verified using a 2100 Bioanalyzer (Agilent Technologies, Santa Clara, CA, USA) and RIN values ranged between 8.1 and 9.6. The enrichment of poly (A) + RNA was performed with 5 μg of total RNA using magnetic beads with Oligo (dT). Following purification, mRNA was fragmented into small pieces. The first cDNA strand was synthesized using random hexamer primers for reverse transcription with cleaved RNA fragments serving as templates. The second strand cDNA was synthesized using RNase H and DNA polymerase I, and the sequencing libraries were constructed following the manufacturer’s instructions. For each cell line (i.e., CHO, MARC-145, and HeLa cells cultivated in sericin-substituted and control media, respectively), one paired-end sequencing library with approximately 300 bp insert size was prepared using the TruSeq RNA-Seq kits (Illumina Inc., San Diego, CA, USA) following the manufacturer’s recommendations. The RNA-seq library was further sequenced with the Illumina HiSeq 2000 platform (Illumina Inc., San Diego, CA, USA). The six sets of raw Illumina sequencing reads of fastq format were trimmed for low-quality reads, ambiguous bases (N), sequencing adapters, primers, and poly (A)/(T) tails.

#### Processing and analysis of RNA-seq data

The trimmed clean reads were aligned to the related reference genome (*Chlorocebus sabaeus* and *Homo sapiens* genomes and annotations were downloaded from Ensembl (http://asia.ensembl.org/info/data/ftp/index.html), and the Chinese hamster (*Cricetulus griseus*) ovary (CHO) cell line genome and annotations were downloaded from CHOgenome (CriGri_1.0, http://chogenome.org/index.php)) employing TopHat2^64^ software with default settings. Calculation of gene expression level and identification of differentially expressed genes (DEGs) between the sericin-substituted and control groups were conducted using Cufflinks 2.0.0^65^. Fragments per kilobase of exon per million fragments mapped (FPKM) were used to normalize RNA-seq fragment counts and estimate the relative abundance of each gene. The Cuffdiff package in Cufflinks 2.0.0^65^ was used to perform pairwise comparisons of the expressions of each gene between cells cultivated in the two conditions and to report DEGs and transcripts. The DEGs were decided based on a *P*-value < 0.05 and at least a 2-fold change between the two FPKMs (higher relative to low one). GO enrichment analyses on DEGs between the two groups were carried out by Web Gene Ontology Annotation Plotting (WEGO)^66^. A Venn diagram was constructed based on the homologs identified by pairwise reciprocal BLAST analysis with e < 10^−5^.

### qRT-PCR validation of DEGs

We performed quantitative reverse transcription-PCR (qRT-PCR) for 10 genes involved in “inhibition of DNA binding” (*ID1, ID2*), “cell cycle progression” (*FN1, MFSD4, MMP1*), and “cell proliferation and migration” (*INHBA, LCN2, CBY1, CXCL12, SCNN1A*). The β-actin gene was chosen as the reference gene^67^. Primers were designed using Oligo software (version 7.60). Three biological replicates and three experimental duplicates were carried out.

### Statistical analysis

For cellular overall survival, cellular DNA content, fluorescence intensity and PDT values, measurement data were obtained with four technical repetitions each time. Data were analyzed by two-tailed paired Student’s *t*-test with an α level of 0.05.

## Additional Information

**Accession Codes:** RNA-seq data are deposited to NCBI SRA (accession number: SRX1722218, SRX1722219, SRX1722229, SRX1722231, SRX1722232 and SRX1722233).

**How to cite this article**: Liu, L. *et al*. Systematic evaluation of sericin protein as a substitute for fetal bovine serum in cell culture. *Sci. Rep.* **6**, 31516; doi: 10.1038/srep31516 (2016).

## Supplementary Material

Supplementary Information
