# Supplementary material for: Systematic evaluation of sericin protein as a substitute for fetal bovine serum in cell culture
Source: Sci Rep. 2016 Aug 17;6:31516. doi: 10.1038/srep31516 (PMC4987615; doi:10.1038/srep31516)
Supplement: Supplementary Information [file srep31516-s1.pdf]

Supporting information

Title:

Systematic evaluation of sericin protein as a substitute for fetal bovine serum in cell culture

Author list:

Liyuan Liu[1, 2, #], Jinhuan Wang[1, #], Shengchang Duan[3, #], Lei Chen[1, 2], Hui Xiang[1, 4] *, Yang Dong[3, *], Wen Wang[1,3,*]

1 State Key Laboratory of Genetic Resources and Evolution, Kunming Institute of Zoology, Chinese Academy of Sciences, Kunming 650223, China.

2 Kunming College of Life Science, University of Chinese Academy of Sciences, Kunming 650223, China.

3 Kunming University of Science and Technology, 727 South Jingming Road, Chenggong District, Kunming 650500, China.

4 South China Normal University, Guangzhou 510631, China.

# These authors contributed equally to this work.

* Corresponding author: wwang@mail.kiz.ac.cn; loyalyang@163.com; xiangh@mail.kiz.ac.cn

Legends for Supplementary Figures and Tables.

Supplementary Fig. S1.

The automated image analyses was performed using CellProfiler software.

Supplementary Fig. S2.

Cell cycle analysis of sericin protein as a substitute for FBS.

Supplementary Fig. S3.

Venn diagram analysis of numbers of DEGs in CHO cells, MARC-145 cells and HeLa cells.

Supplementary Fig. S4.

qRT-PCR validation of differentially expressed genes.

Supplemental Table S1.

List of the top 10 significantly up-regulated and down-regulated genes of CHO cells under the condition of sericin- substituted culture media compared to the control medium.

Supplemental Table S2.

List of the top 10 significantly up-regulated and down-regulated genes of HeLa cells under the condition of sericin- substituted culture media compared to the control medium.

Supplemental Table S3.

List of the top 10 significantly up-regulated and down-regulated genes of MARC-145 cells under the condition of sericin- substituted culture media compared to the control medium.

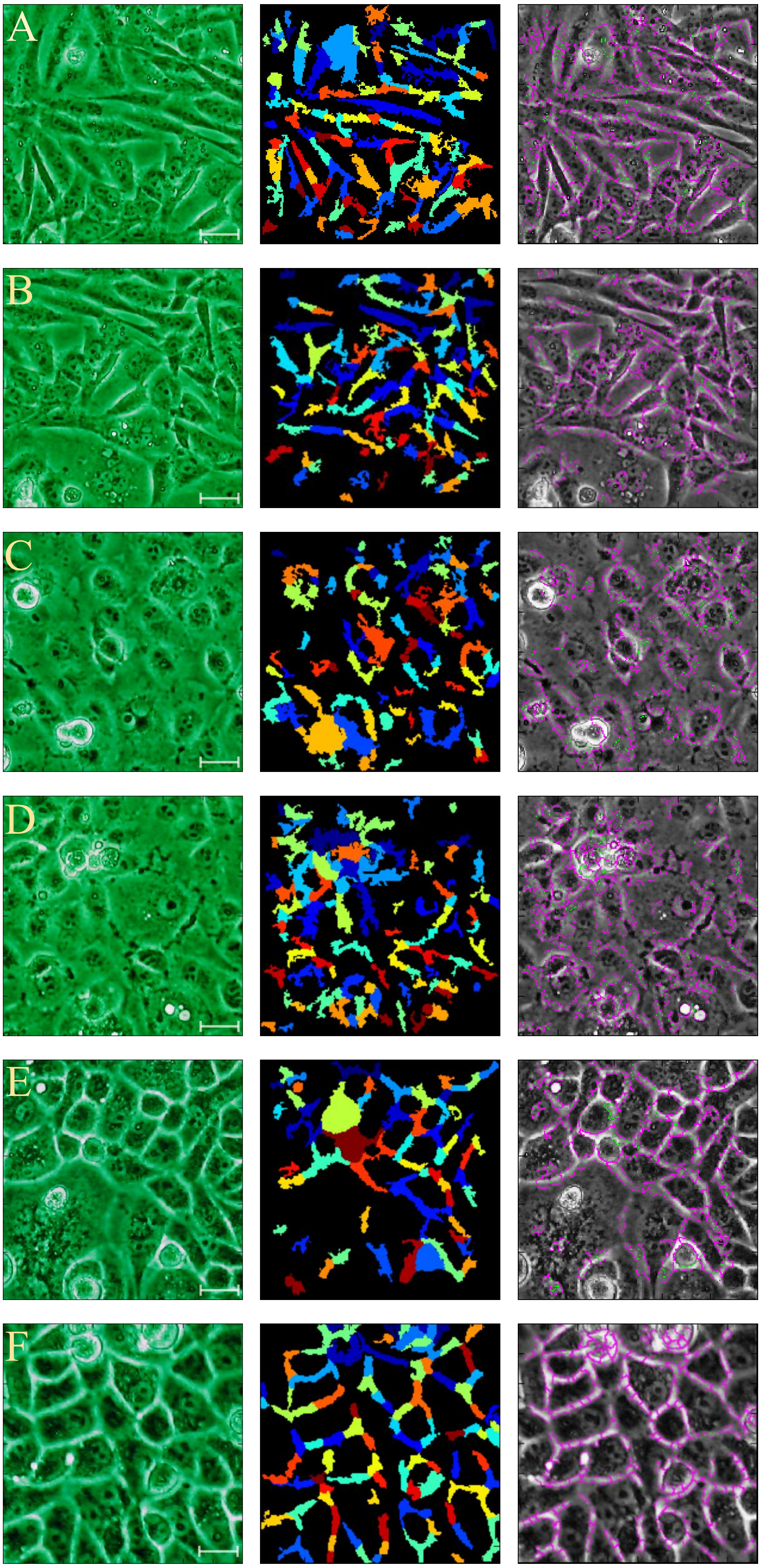

Supplementary Fig. S1 **The automated image analyses was performed using CellProfiler software.** The figure shows the fluorescence micrographs (left lane), the cell and intercellular space from the image (middle lane), the cell size and outline (right lane). The automated image analyses showed that no significant difference of cell morphology was observed in cells cultivated in the sericin-substituted medium. A, B. CHO cells; C, D. MARC-145 cells; E, F. HeLa cells. A, C, E. control medium (10% FBS); B, D, F. sericin-substituted medium (30 μg/ml sericin protein). Scale bars represent 40 μm.

(**A**)

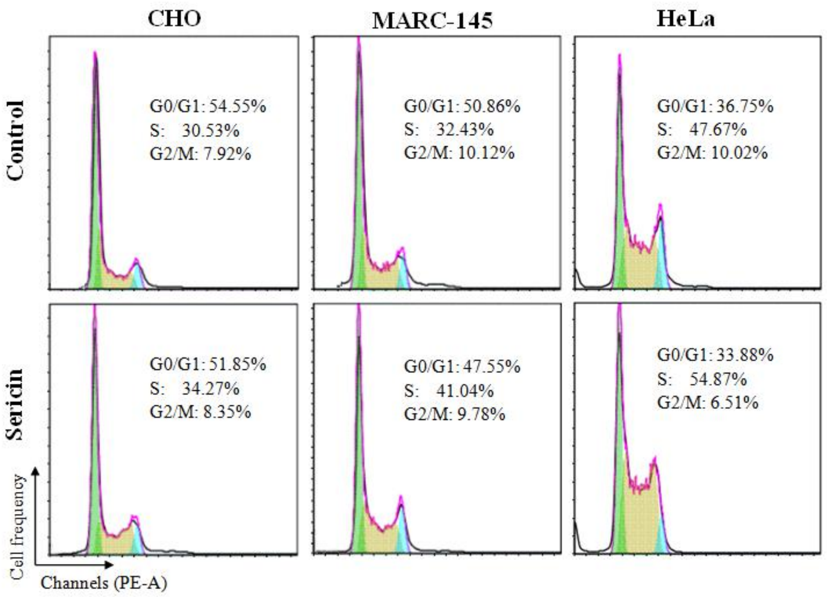

(**B**)

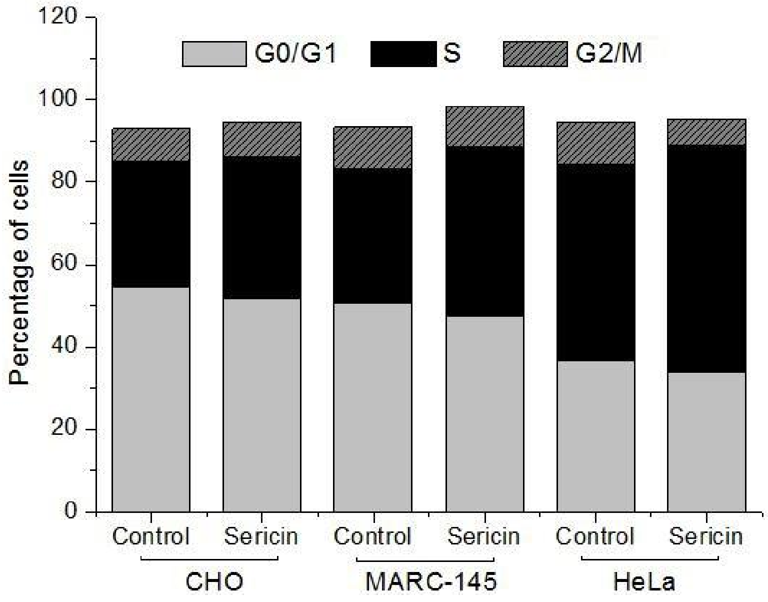

Supplementary Fig. S2 **Cell cycle analysis of sericin protein as a substitute for FBS.** (A) Representative images of flow cytometry analysis carried out at 80-90% cell confluence. (B) The statistic results of flow cytometry. Cell cycles of the three cell lines were significantly aggregation in the S phase in the sericin-substituted medium. The number of cells in the G0/G1-, S- or G2/M-phase is given as percentages of the total cell population.

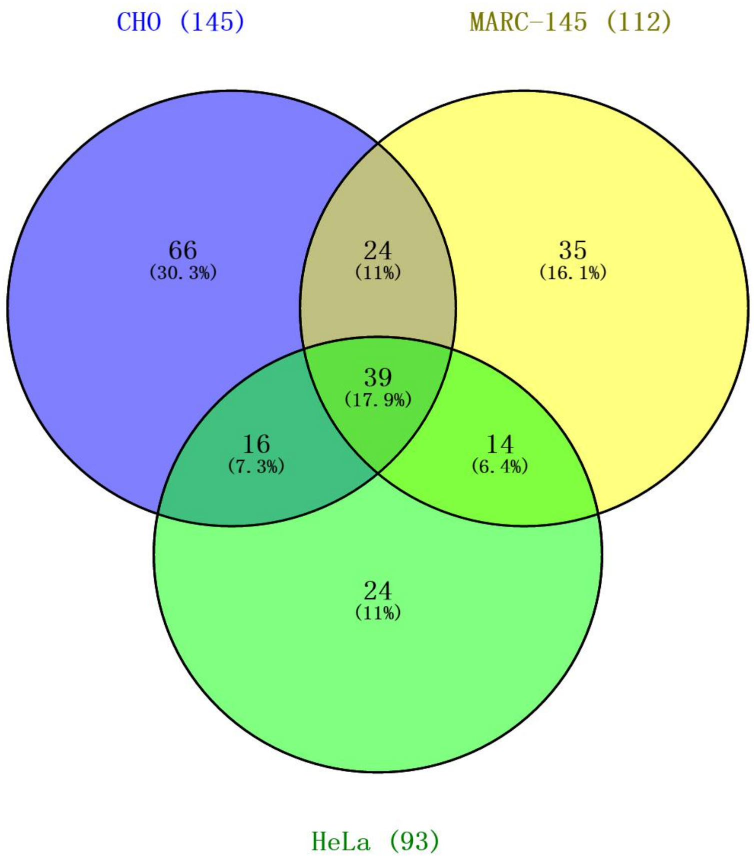

Supplementary Fig. S3 **Venn diagram analysis of numbers of DEGs in CHO cells,MARC-145 cells and HeLa cells.** The high overlap of the DEGs suggested that the analysis of RNA-seq was reliable and consistent.

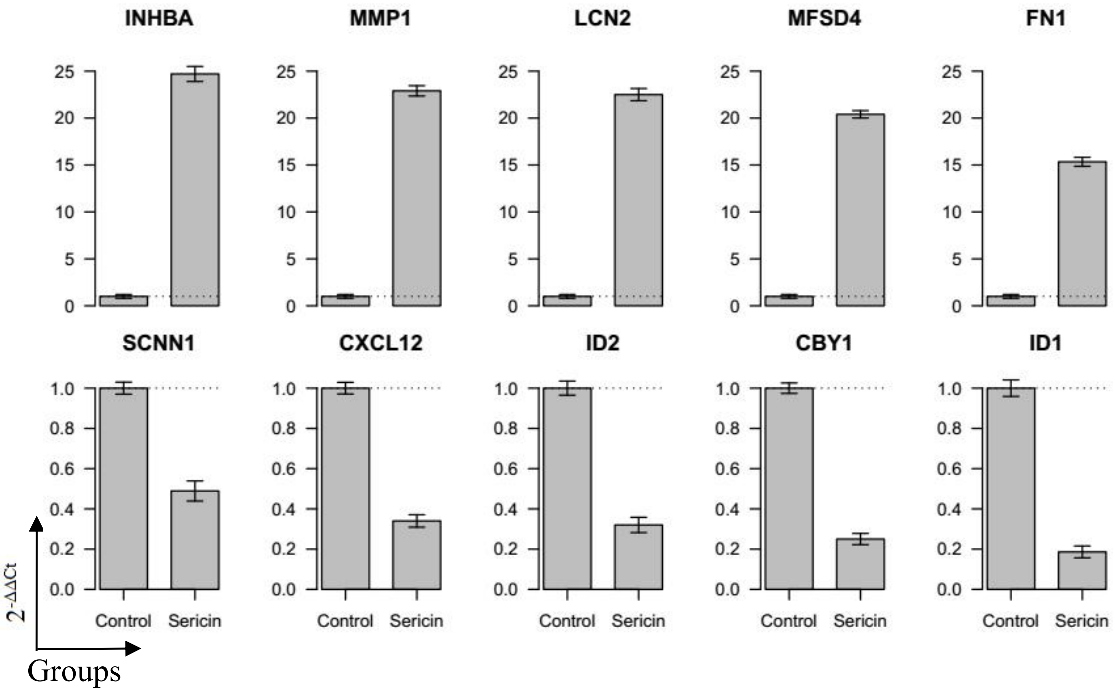

Supplementary Fig. S4 **qRT-PCR validation of differentially expressed genes.** Ten DGEs of MACR-145 cells were selected for validation by qRT-PCR and all of them showed consistent trends between RNA-seq and qRT-PCR trials. Y-axis represents the relative expression value ($2^{-\Delta\Delta Ct}$, normalized to mean in control) compared to reference gene β-actin (*ACTB*).

Supplemental tables

Supplemental Table S1 List of the top 10 significantly up-regulated and down-regulated genes of CHO cells under the condition of sericin- substituted culture media compared to the control medium

Gene	Locus position	Gene description	FPKM-FBS	FPKM-Sericin	log2 fold change	Test statistic	p-value	q-value
Isg15	NW_003614798.1:5953-7313	ISG15 ubiquitin-like modifier	3.3283	133.1580	5.3222	-11.8164	9.5405E-04	4.9526E-02
LOC100762031	NW_003613905.1:465300-492621	interferon-induced GTP-binding protein Mx2	2.6582	30.3176	3.5116	-8.9124	8.5987E-04	4.6253E-02
Cxcl1	NW_003614806.1:398742-400542	chemokine (C-X-C motif) ligand 1	15.7357	167.7210	3.4139	-9.6718	8.3097E-05	9.5344E-03
Hmgcs1	NW_003614106.1:1047105-1066853	3-hydroxy-3-methylglutaryl-CoA synthase 1 (soluble)	45.4773	456.0640	3.3260	-9.4111	1.1293E-11	1.2076E-08
Lgr6	NW_003613706.1:834953-952353	leucine-rich repeat containing G protein-coupled receptor 6	2.0008	15.6909	2.9712	-3.9513	7.7740E-05	9.1154E-03
Mmp10	NW_003613612.1:796750-805658	matrix metallopeptidase 10 (stromelysin 2)	23.4823	162.6640	2.7923	-8.9289	9.0468E-08	3.4192E-05
Nfkbiz	NW_003614243.1:760974-790549	nuclear factor of kappa light polypeptide gene enhancer in B-cells inhibitor, zeta	2.0208	13.8635	2.7783	-6.6093	3.8621E-11	3.9711E-08
Filip1l	NW_003614098.1:388950-659318	filamin A interacting protein 1-like	1.7792	12.1845	2.7757	-5.2082	1.9068E-07	5.9971E-05
Idi1	NW_003613580.1:8255356-8264801	isopentenyl-diphosphate delta isomerase 1	26.3124	176.9630	2.7496	-8.6423	1.5987E-13	2.2495E-10
Mvd	NW_003613583.1:1094985-1105448	mevalonate (diphospho) decarboxylase	21.1287	140.5160	2.7335	-8.3155	3.6389E-06	6.8995E-04
Id1	NW_003613890.1:1317086-1318255	inhibitor of DNA binding 1, dominant negative helix-loop-helix protein	80.5077	6.9470	-3.5347	9.0409	1.2927E-09	8.0367E-07
Vnn1	NW_003614986.1:179594-195907	vanin 1	18.9879	2.0043	-3.2439	7.7512	9.1038E-15	1.6226E-11
Ccr7	NW_003614892.1:402867-413641	chemokine (C-C motif) receptor 7	15.2009	1.7571	-3.1129	7.6339	2.2871E-14	3.5966E-11
Plet1	NW_003613694.1:1391336-1409661	placenta expressed transcript 1	21.4262	3.7662	-2.5082	6.8474	7.5209E-12	8.7419E-09
S100a4	NW_003613854.1:1799415-1801809	S100 calcium binding protein A4	80.7505	14.8139	-2.4465	4.9562	7.1876E-07	1.8476E-04
LOC100763954	NW_003613842.1:1291506-1297692	amiloride-sensitive amine oxidase [copper-containing]	10.6956	1.9695	-2.4411	6.0683	1.2927E-09	8.0367E-07
Ankrd1	NW_003613711.1:1386445-1420393	ankyrin repeat domain 1 (cardiac muscle)	6.7338	1.3917	-2.2746	4.6563	3.2200E-06	6.3297E-04
Hmox1	NW_003615294.1:116406-127568	heme oxygenase (decycling) 1	395.1640	89.2680	-2.1462	7.0395	1.9291E-12	2.3442E-09
Id3	NW_003614105.1:149913-151477	inhibitor of DNA binding 3, dominant negative helix-loop-helix protein	127.1150	30.1295	-2.0769	6.3128	2.7400E-10	2.1545E-07
Tspan7	NW_003613590.1:667131-688234	tetraspanin 7	4.9229	1.2892	-1.9331	3.8949	9.8256E-05	1.0753E-02

Supplemental Table S2 List of the top 10 significantly up-regulated and down-regulated genes of HeLa cells under the condition of sericin- substituted culture media compared to the control medium

Gene	Locus position	Gene description	FPKM-FBS	FPKM-Sericin	log2 fold change	Test statistic	p-value	q-value
RMRP	9:35657750-35658018	RNA component of mitochondrial RNA processing endoribonuclease	109.7240	632.8730	2.5280	-9.2135	2.4184E-10	2.3113E-08
TXNIP	1:145992434-145996600	thioredoxin interacting protein	2.9205	10.4563	1.8401	-6.4076	1.4784E-10	2.2327E-07
ABCA13	7:48171457-48647496	ATP-binding cassette, sub-family A (ABC1), member 13	1.7113	4.6117	1.4302	-4.5949	4.3286E-06	2.6634E-03
HDAC5	17:44076745-44123702	histone deacetylase 5	8.9266	21.2988	1.2546	-3.9187	8.9037E-05	2.5950E-02
ACSS2	20:34844719-34927962	acyl-CoA synthetase short-chain family member 2	41.9626	98.0355	1.2242	-4.0822	4.4608E-05	1.6110E-02
CNTNAP1	17:42678888-42699814	contactin associated protein 1	2.4288	5.6122	1.2083	-3.9371	8.2487E-05	2.4915E-02
NR4A2	2:156324431-156342348	nuclear receptor subfamily 4, group A, member 2	9.0408	20.6913	1.1945	-4.7671	1.8692E-06	1.2614E-03
SCD	10:100347123-100364834	stearoyl-CoA desaturase (delta-9-desaturase)	191.6800	416.8230	1.1207	-4.5259	6.0132E-06	3.4447E-03
MOB3B	9:27325208-27530659	MOB kinase activator 3B	1.7812	3.7824	1.0864	-4.5321	5.8390E-06	3.4447E-03
NR1D1	17:40019096-40100725	nuclear receptor subfamily 1, group D, member 1	13.5252	28.1467	1.0573	-3.7531	6.6900E-07	4.0870E-02
CYP1A1	15:74719541-74725959	cytochrome P450, family 1, subfamily A, polypeptide 1	5.7078	1.1697	-2.2868	7.0436	1.8738E-12	3.1130E-09
ID1	20:31605282-31606515	inhibitor of DNA binding 1, dominant negative helix-loop-helix protein	188.3430	39.0386	-2.2704	12.1324	9.4070E-06	1.9739E-07
MIR23A	19:13795459-13842799	microRNA 23a	19.4648	6.1407	-1.6644	5.6526	1.5802E-08	2.0193E-05
MIR27A	19:13795459-13842899	microRNA 27a	19.4648	6.1407	-1.6644	5.6526	1.5802E-08	2.0193E-05
ID3	1:23557917-23559794	inhibitor of DNA binding 3, dominant negative helix-loop-helix protein	142.7590	48.2539	-1.5649	9.0187	3.3478E-10	4.3113E-06
SERPINE1	7:101126653-101140192	serpin peptidase inhibitor, clade E , member 1	35.2691	12.3745	-1.5110	7.5259	5.2403E-14	1.0882E-10
ID2	2:8666635-8684453	inhibitor of DNA binding 2, dominant negative helix-loop-helix protein	10.4508	3.7016	-1.4974	4.0152	5.9407E-05	1.9739E-02
HMOX1	22:35380360-35394214	heme oxygenase 1	13.8197	5.2937	-1.3844	5.4801	4.2500E-08	4.7070E-05
PDK4	7:95583498-95596491	pyruvate dehydrogenase kinase, isozyme 4	29.5967	12.7988	-1.2094	5.1725	2.3102E-07	1.8911E-04
B4GALT4	3:119211731-119290666	UDP-Gal:betaGlcNAc beta 1,4- galactosyltransferase, polypeptide 4	24.1237	10.6416	-1.1807	4.3590	1.3064E-05	6.0285E-03

Supplemental Table S3 List of the top 10 significantly up-regulated and down-regulated genes of MARC-145 cells under the condition of sericin- substituted culture media compared to the control medium

Gene	Locus position	Gene description	FPKM-FBS	FPKM-Sericin	log2 fold change	Test statistic	p-value	q-value
INHBA	21:16936080-16951547	inhibin, beta A	3.5595	51.0573	3.8424	-7.4084	1.2790E-13	3.0947E-10
LCN2	12:9976613-9980870	lipocalin 2	1.7995	20.5503	3.5135	-5.3093	1.1004E-07	8.5890E-05
MFSD4	25:23741893-23774451	major facilitator superfamily domain containing 4	1.4822	14.8479	3.3244	-3.6108	3.0527E-04	3.1839E-02
MMP1	1:94167205-94176767	matrix metallopeptidase 1 (interstitial collagenase)	38.1203	343.0570	3.1698	-9.6443	1.8300E-05	1.1705E-03
FN1	10:101176788-101254716	fibronectin 1	4.8266	36.4082	2.9152	-8.2642	2.2205E-16	6.7160E-13
PLAT	8:40163890-40196555	plasminogen activator, tissue	1.2989	7.1571	2.4621	-5.2772	1.3119E-07	9.9199E-05
SFRP1	8:39243779-39289088	secreted frizzled-related protein 1	4.9055	26.6830	2.4435	-4.8391	1.3046E-06	6.4424E-04
WFDC3	2:18098204-18115049	WAP four-disulfide core domain 3	12.8112	67.7871	2.4036	-4.3964	1.1008E-05	3.1711E-03
HES1	15:88220082-88223243	hes family bHLH transcription factor 1	16.1660	81.0575	2.3260	-6.0805	1.1979E-09	1.6104E-06
LTBP1	14:74063334-74548875	latent transforming growth factor beta binding protein 1	1.2246	5.7556	2.2326	-4.9550	7.2334E-07	3.8049E-04
ID1	2:35997972-36010017	inhibitor of DNA binding 1, dominant negative helix-loop-helix protein	197.8190	17.0491	-3.5364	10.7075	3.3481E-04	3.3616E-02
CBY1	19:21318142-21344765	chibby homolog 1	42.1451	4.0154	-3.3917	4.2159	2.4878E-05	5.5594E-03
CXCL12	9:40005582-40020529	chemokine (C-X-C motif) ligand 12	68.2679	10.2098	-2.7413	3.5146	4.4049E-04	3.8758E-02
ID2	14:98961545-98964882	inhibitor of DNA binding 2, dominant negative helix-loop-helix protein	100.8640	15.6893	-2.6846	8.4064	0.0000E+00	0.0000E+00
SCNN1A	11:6383955-6417786	sodium channel, non-voltage-gated 1 alpha subunit	9.9745	1.7986	-2.4714	3.9139	9.0802E-05	1.3358E-02
HMOX1	19:18144544-18157971	heme oxygenase (decycling) 1	254.4780	52.6479	-2.2731	8.0190	1.1102E-15	2.9849E-12
VGF	28:12017435-12021586	VGF nerve growth factor inducible	38.0295	7.9330	-2.2612	6.3429	2.2555E-10	3.4110E-07
STYK1	11:10419663-10475476	serine/threonine/tyrosine kinase 1	7.0743	1.5691	-2.1726	3.8893	1.0052E-04	1.4335E-02
BMP4	24:31097537-31104746	bone morphogenetic protein 4	59.1441	13.9183	-2.0873	5.7618	8.3203E-09	9.1512E-06
ARRB2	16:4191171-4202385	arrestin, beta 2	4.3486	1.1044	-1.9772	3.6472	2.6508E-04	2.9023E-02
